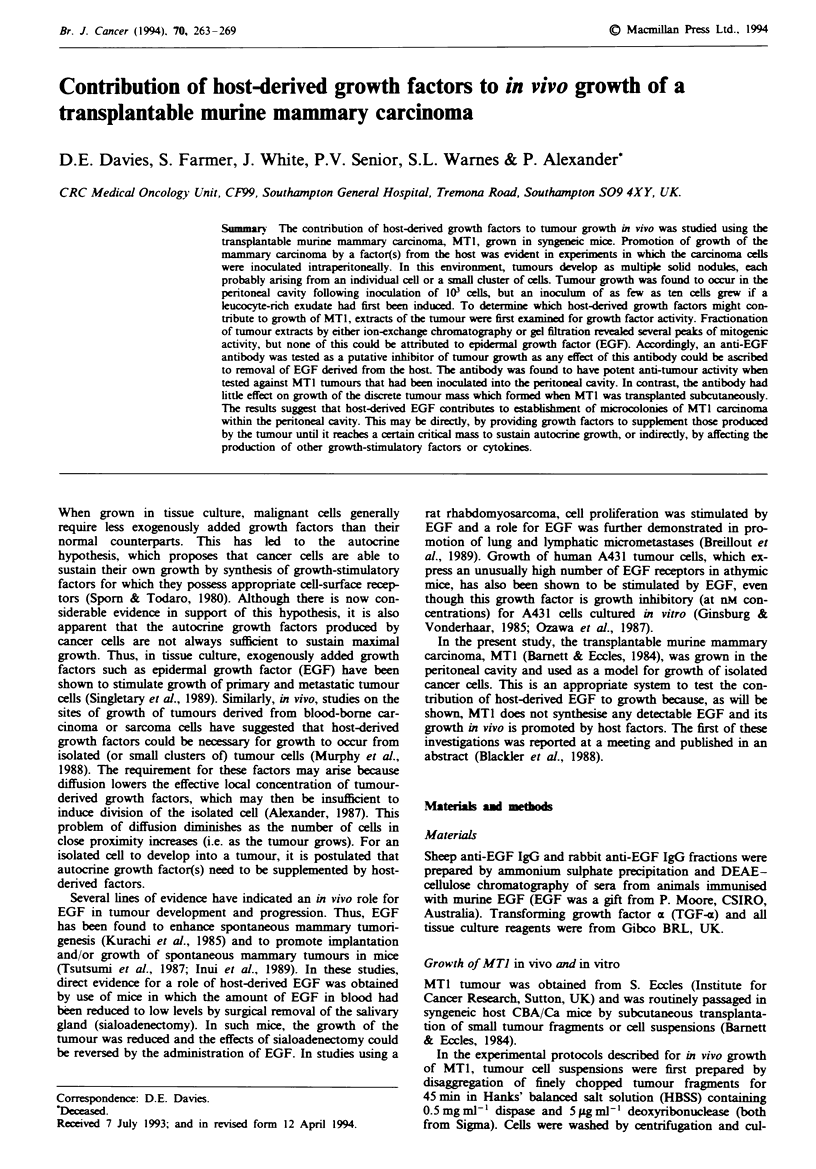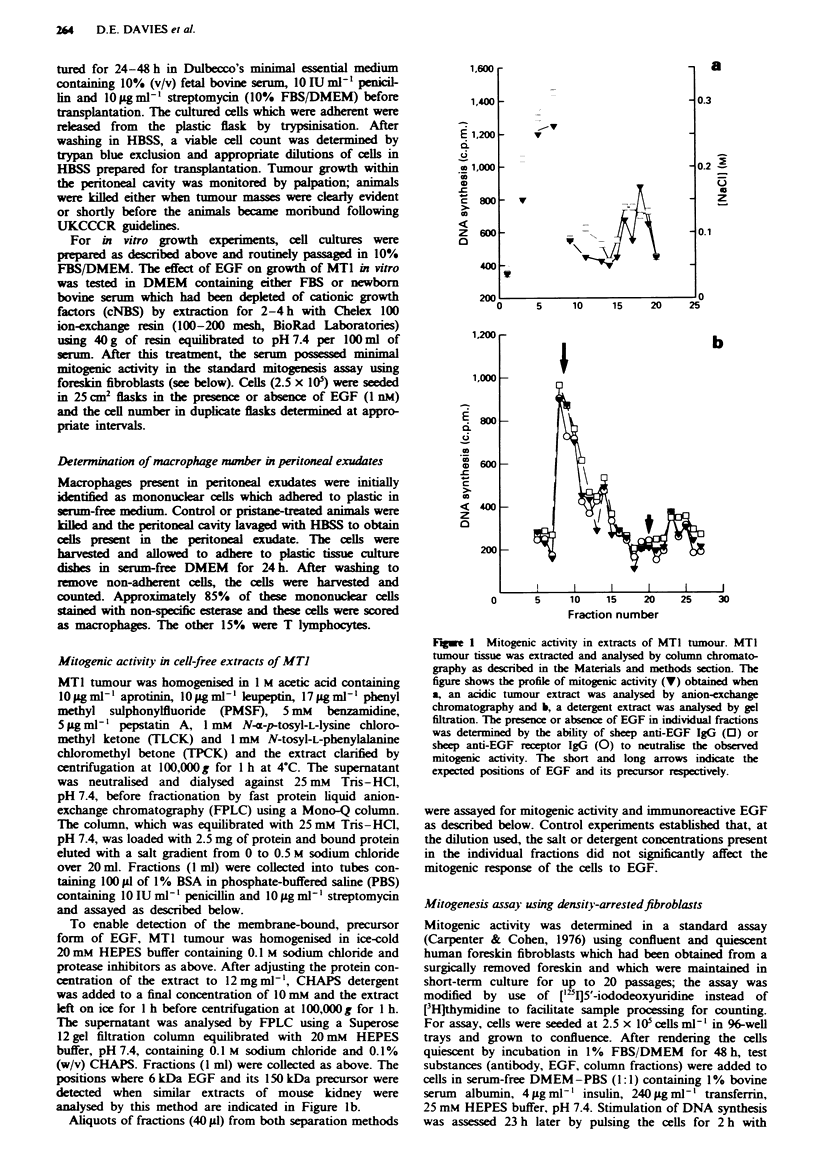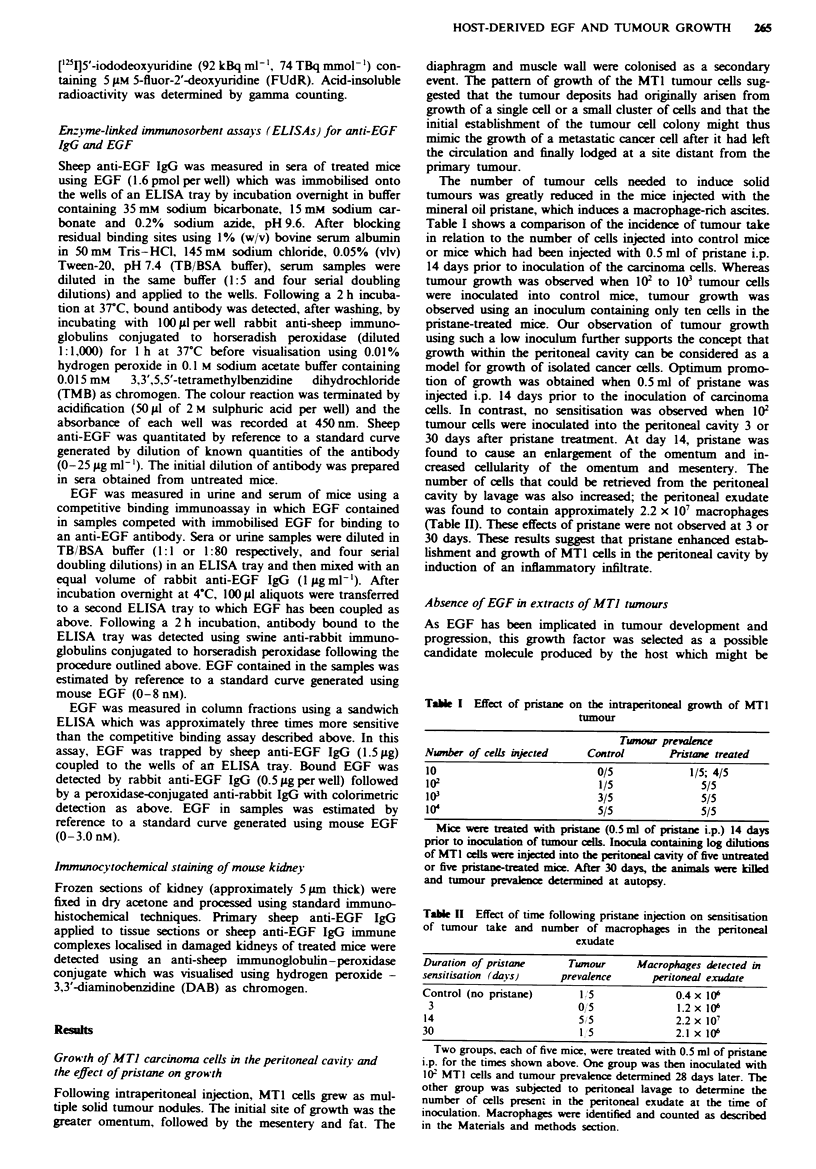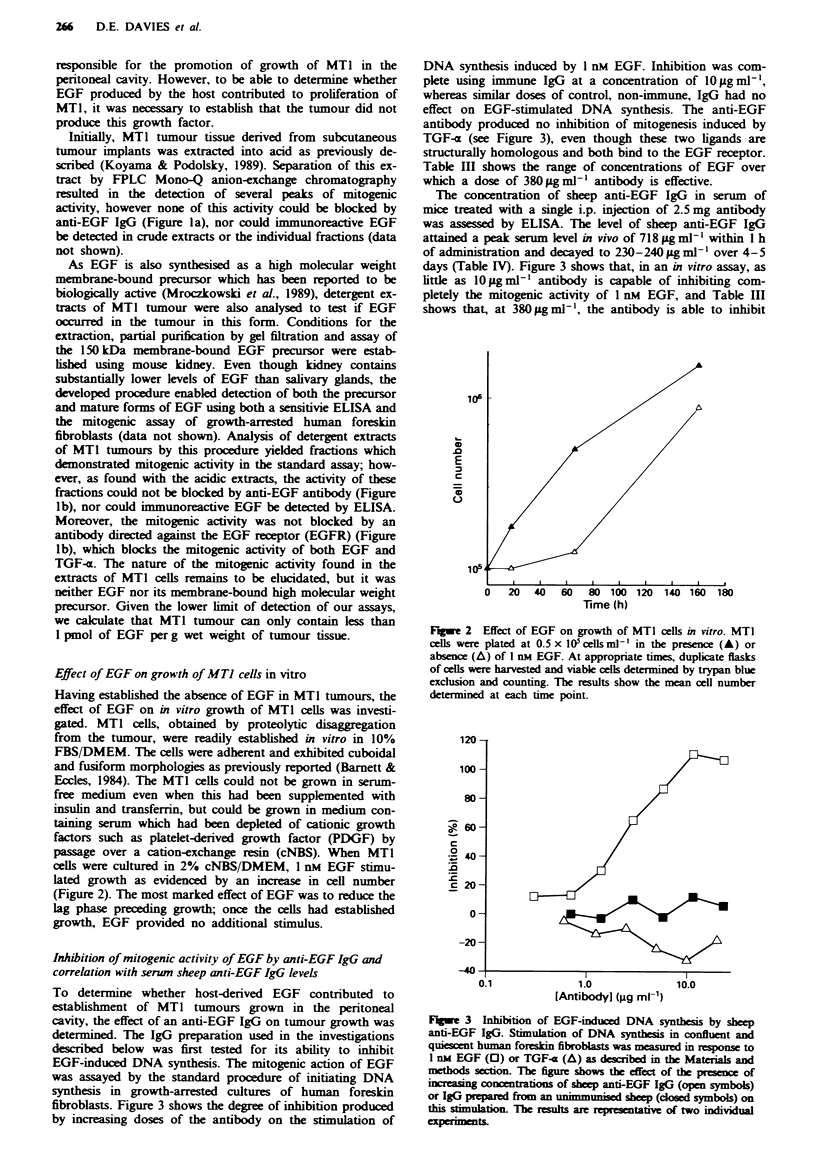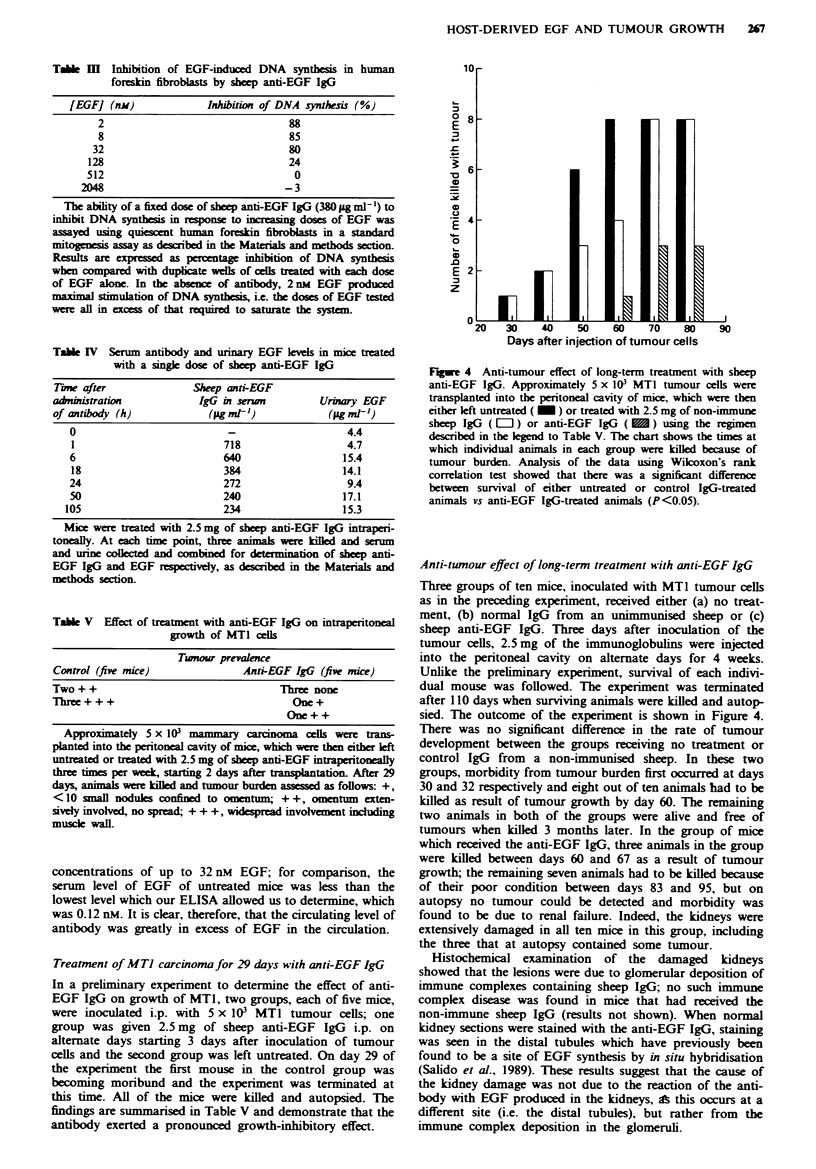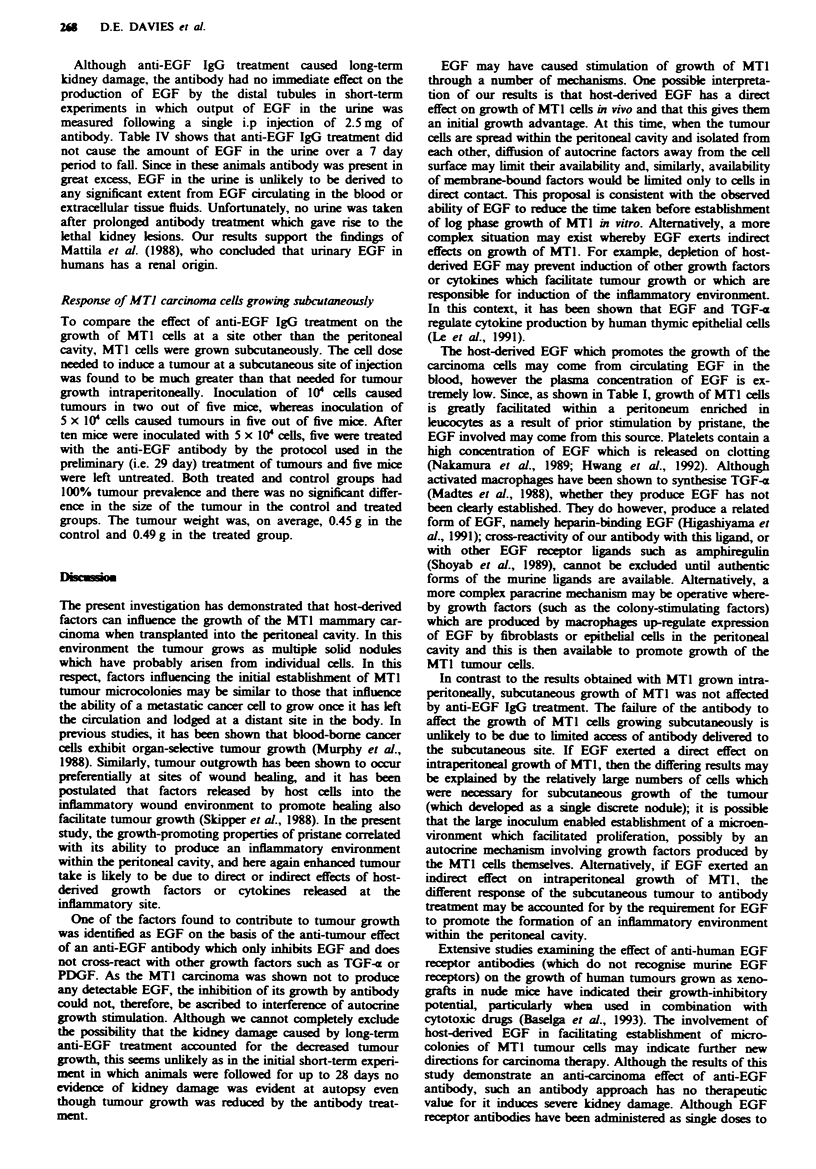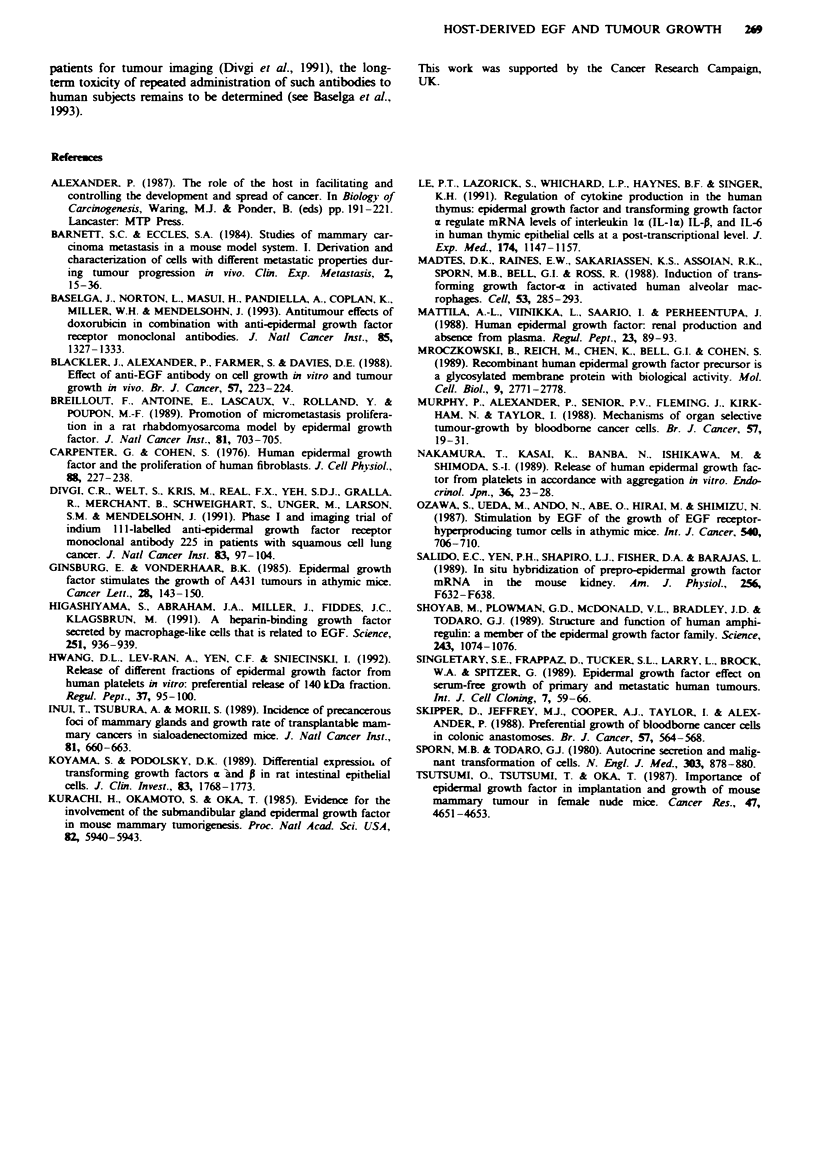# Contribution of host-derived growth factors to in vivo growth of a transplantable murine mammary carcinoma.

**DOI:** 10.1038/bjc.1994.290

**Published:** 1994-08

**Authors:** D. E. Davies, S. Farmer, J. White, P. V. Senior, S. L. Warnes, P. Alexander

**Affiliations:** CRC Medical Oncology Unit, Southampton General Hospital, UK.

## Abstract

The contribution of host-derived growth factors to tumour growth in vivo was studied using the transplantable murine mammary carcinoma, MT1, grown in syngeneic mice. Promotion of growth of the mammary carcinoma by a factor(s) from the host was evident in experiments in which the carcinoma cells were inoculated intraperitoneally. In this environment, tumours develop as multiple solid nodules, each probably arising from an individual cell or a small cluster of cells. Tumour growth was found to occur in the peritoneal cavity following inoculation of 10(3) cells, but an inoculum of as few as ten cells grew if a leucocyte-rich exudate had first been induced. To determine which host-derived growth factors might contribute to growth of MT1, extracts of the tumour were first examined for growth factor activity. Fractionation of tumour extracts by either ion-exchange chromatography or gel filtration revealed several peaks of mitogenic activity, but none of this could be attributed to epidermal growth factor (EGF). Accordingly, an anti-EGF antibody was tested as a putative inhibitor of tumour growth as any effect of this antibody could be ascribed to removal of EGF derived from the host. The antibody was found to have potent anti-tumour activity when tested against MT1 tumours that had been inoculated into the peritoneal cavity. In contrast, the antibody had little effect on growth of the discrete tumour mass which formed when MT1 was transplanted subcutaneously. The results suggest that host-derived EGF contributes to establishment of microcolonies of MT1 carcinoma within the peritoneal cavity. This may be directly, by providing growth factors to supplement those produced by the tumour until it reaches a certain critical mass to sustain autocrine growth, or indirectly, by affecting the production of other growth-stimulatory factors or cytokines.


Br. J. Cancer (1994), 70, 263-269 © Macmillan Press Ltd., 1994

### Contribution of host-derived growth factors to *in vivo* growth of a transplantable murine mammary carcinoma

D.E. Davies, S. Farmer, J. White, P.V. Senior, S.L. Warnes & P. Alexander*

*CRC Medical Oncology Unit, CF99, Southampton General Hospital, Tremona Road, Southampton SO9 4XY, UK.*

**Summary** The contribution of host-derived growth factors to tumour growth *in vivo* was studied using the transplantable murine mammary carcinoma, MT1, grown in syngeneic mice. Promotion of growth of the mammary carcinoma by a factor(s) from the host was evident in experiments in which the carcinoma cells were inoculated intraperitoneally. In this environment, tumours develop as multiple solid nodules, each probably arising from an individual cell or a small cluster of cells. Tumour growth was found to occur in the peritoneal cavity following inoculation of $10^3$ cells, but an inoculum of as few as ten cells grew if a leucocyte-rich exudate had first been induced. To determine which host-derived growth factors might contribute to growth of MT1, extracts of the tumour were first examined for growth factor activity. Fractionation of tumour extracts by either ion-exchange chromatography or gel filtration revealed several peaks of mitogenic activity, but none of this could be attributed to epidermal growth factor (EGF). Accordingly, an anti-EGF antibody was tested as a putative inhibitor of tumour growth as any effect of this antibody could be ascribed to removal of EGF derived from the host. The antibody was found to have potent anti-tumour activity when tested against MT1 tumours that had been inoculated into the peritoneal cavity. In contrast, the antibody had little effect on growth of the discrete tumour mass which formed when MT1 was transplanted subcutaneously. The results suggest that host-derived EGF contributes to establishment of microcolonies of MT1 carcinoma within the peritoneal cavity. This may be directly, by providing growth factors to supplement those produced by the tumour until it reaches a certain critical mass to sustain autocrine growth, or indirectly, by affecting the production of other growth-stimulatory factors or cytokines.

When grown in tissue culture, malignant cells generally require less exogenously added growth factors than their normal counterparts. This has led to the autocrine hypothesis, which proposes that cancer cells are able to sustain their own growth by synthesis of growth-stimulatory factors for which they possess appropriate cell-surface receptors (Sporn & Todaro, 1980). Although there is now considerable evidence in support of this hypothesis, it is also apparent that the autocrine growth factors produced by cancer cells are not always sufficient to sustain maximal growth. Thus, in tissue culture, exogenously added growth factors such as epidermal growth factor (EGF) have been shown to stimulate growth of primary and metastatic tumour cells (Singletary *et al.*, 1989). Similarly, *in vivo*, studies on the sites of growth of tumours derived from blood-borne carcinoma or sarcoma cells have suggested that host-derived growth factors could be necessary for growth to occur from isolated (or small clusters of) tumour cells (Murphy *et al.*, 1988). The requirement for these factors may arise because diffusion lowers the effective local concentration of tumour-derived growth factors, which may then be insufficient to induce division of the isolated cell (Alexander, 1987). This problem of diffusion diminishes as the number of cells in close proximity increases (i.e. as the tumour grows). For an isolated cell to develop into a tumour, it is postulated that autocrine growth factor(s) need to be supplemented by host-derived factors.

Several lines of evidence have indicated an *in vivo* role for EGF in tumour development and progression. Thus, EGF has been found to enhance spontaneous mammary tumorigenesis (Kurachi *et al.*, 1985) and to promote implantation and/or growth of spontaneous mammary tumours in mice (Tsutsumi *et al.*, 1987; Inui *et al.*, 1989). In these studies, direct evidence for a role of host-derived EGF was obtained by use of mice in which the amount of EGF in blood had been reduced to low levels by surgical removal of the salivary gland (sialoadenectomy). In such mice, the growth of the tumour was reduced and the effects of sialoadenectomy could be reversed by the administration of EGF. In studies using a rat rhabdomyosarcoma, cell proliferation was stimulated by EGF and a role for EGF was further demonstrated in promotion of lung and lymphatic micrometastases (Breillout *et al.*, 1989). Growth of human A431 tumour cells, which express an unusually high number of EGF receptors in athymic mice, has also been shown to be stimulated by EGF, even though this growth factor is growth inhibitory (at nM concentrations) for A431 cells cultured *in vitro* (Ginsburg & Vonderhaar, 1985; Ozawa *et al.*, 1987).

In the present study, the transplantable murine mammary carcinoma, MT1 (Barnett & Eccles, 1984), was grown in the peritoneal cavity and used as a model for growth of isolated cancer cells. This is an appropriate system to test the contribution of host-derived EGF to growth because, as will be shown, MT1 does not synthesise any detectable EGF and its growth *in vivo* is promoted by host factors. The first of these investigations was reported at a meeting and published in an abstract (Blackler *et al.*, 1988).

### Materials and methods

### *Materials*

Sheep anti-EGF IgG and rabbit anti-EGF IgG fractions were prepared by ammonium sulphate precipitation and DEAE-cellulose chromatography of sera from animals immunised with murine EGF (EGF was a gift from P. Moore, CSIRO, Australia). Transforming growth factor α (TGF-α) and all tissue culture reagents were from Gibco BRL, UK.

### *Growth of MT1* in vivo *and* in vitro

MT1 tumour was obtained from S. Eccles (Institute for Cancer Research, Sutton, UK) and was routinely passaged in syngeneic host CBA/Ca mice by subcutaneous transplantation of small tumour fragments or cell suspensions (Barnett & Eccles, 1984).

In the experimental protocols described for *in vivo* growth of MT1, tumour cell suspensions were first prepared by disaggregation of finely chopped tumour fragments for 45 min in Hanks' balanced salt solution (HBSS) containing 0.5 mg $ml^{-1}$ dispase and 5 μg $ml^{-1}$ deoxyribonuclease (both from Sigma). Cells were washed by centrifugation and cul-

Correspondence: D.E. Davies.
*Deceased.
Received 7 July 1993; and in revised form 12 April 1994.

tured for 24–48 h in Dulbecco's minimal essential medium containing 10% (v/v) fetal bovine serum, 10 IU ml$^{-1}$ penicillin and 10 μg ml$^{-1}$ streptomycin (10% FBS/DMEM) before transplantation. The cultured cells which were adherent were released from the plastic flask by trypsinisation. After washing in HBSS, a viable cell count was determined by trypan blue exclusion and appropriate dilutions of cells in HBSS prepared for transplantation. Tumour growth within the peritoneal cavity was monitored by palpation; animals were killed either when tumour masses were clearly evident or shortly before the animals became moribund following UKCCCR guidelines.

For *in vitro* growth experiments, cell cultures were prepared as described above and routinely passaged in 10% FBS/DMEM. The effect of EGF on growth of MT1 *in vitro* was tested in DMEM containing either FBS or newborn bovine serum which had been depleted of cationic growth factors (cNBS) by extraction for 2–4 h with Chelex 100 ion-exchange resin (100–200 mesh, BioRad Laboratories) using 40 g of resin equilibrated to pH 7.4 per 100 ml of serum. After this treatment, the serum possessed minimal mitogenic activity in the standard mitogenesis assay using foreskin fibroblasts (see below). Cells (2.5 × 10$^5$) were seeded in 25 cm$^2$ flasks in the presence or absence of EGF (1 nM) and the cell number in duplicate flasks determined at appropriate intervals.

### *Determination of macrophage number in peritoneal exudates*

Macrophages present in peritoneal exudates were initially identified as mononuclear cells which adhered to plastic in serum-free medium. Control or pristane-treated animals were killed and the peritoneal cavity lavaged with HBSS to obtain cells present in the peritoneal exudate. The cells were harvested and allowed to adhere to plastic tissue culture dishes in serum-free DMEM for 24 h. After washing to remove non-adherent cells, the cells were harvested and counted. Approximately 85% of these mononuclear cells stained with non-specific esterase and these cells were scored as macrophages. The other 15% were T lymphocytes.

### *Mitogenic activity in cell-free extracts of MT1*

MT1 tumour was homogenised in 1 M acetic acid containing 10 μg ml$^{-1}$ aprotinin, 10 μg ml$^{-1}$ leupeptin, 17 μg ml$^{-1}$ phenyl methyl sulphonylfluoride (PMSF), 5 mM benzamidine, 5 μg ml$^{-1}$ pepstatin A, 1 mM *N*-α-*p*-tosyl-L-lysine chloromethyl ketone (TLCK) and 1 mM *N*-tosyl-L-phenylalanine chloromethyl betone (TPCK) and the extract clarified by centrifugation at 100,000 *g* for 1 h at 4°C. The supernatant was neutralised and dialysed against 25 mM Tris–HCl, pH 7.4, before fractionation by fast protein liquid anion-exchange chromatography (FPLC) using a Mono-Q column. The column, which was equilibrated with 25 mM Tris–HCl, pH 7.4, was loaded with 2.5 mg of protein and bound protein eluted with a salt gradient from 0 to 0.5 M sodium chloride over 20 ml. Fractions (1 ml) were collected into tubes containing 100 μl of 1% BSA in phosphate-buffered saline (PBS) containing 10 IU ml$^{-1}$ penicillin and 10 μg ml$^{-1}$ streptomycin and assayed as described below.

To enable detection of the membrane-bound, precursor form of EGF, MT1 tumour was homogenised in ice-cold 20 mM HEPES buffer containing 0.1 M sodium chloride and protease inhibitors as above. After adjusting the protein concentration of the extract to 12 mg ml$^{-1}$, CHAPS detergent was added to a final concentration of 10 mM and the extract left on ice for 1 h before centrifugation at 100,000 *g* for 1 h. The supernatant was analysed by FPLC using a Superose 12 gel filtration column equilibrated with 20 mM HEPES buffer, pH 7.4, containing 0.1 M sodium chloride and 0.1% (w/v) CHAPS. Fractions (1 ml) were collected as above. The positions where 6 kDa EGF and its 150 kDa precursor were detected when similar extracts of mouse kidney were analysed by this method are indicated in Figure 1b.

Aliquots of fractions (40 μl) from both separation methods

**Figure 1** Mitogenic activity in extracts of MT1 tumour. MT1 tumour tissue was extracted and analysed by column chromatography as described in the Materials and methods section. The figure shows the profile of mitogenic activity (▼) obtained when **a**, an acidic tumour extract was analysed by anion-exchange chromatography and **b**, a detergent extract was analysed by gel filtration. The presence or absence of EGF in individual fractions was determined by the ability of sheep anti-EGF IgG (□) or sheep anti-EGF receptor IgG (○) to neutralise the observed mitogenic activity. The short and long arrows indicate the expected positions of EGF and its precursor respectively.

were assayed for mitogenic activity and immunoreactive EGF as described below. Control experiments established that, at the dilution used, the salt or detergent concentrations present in the individual fractions did not significantly affect the mitogenic response of the cells to EGF.

### *Mitogenesis assay using density-arrested fibroblasts*

Mitogenic activity was determined in a standard assay (Carpenter & Cohen, 1976) using confluent and quiescent human foreskin fibroblasts which had been obtained from a surgically removed foreskin and which were maintained in short-term culture for up to 20 passages; the assay was modified by use of [$^{125}$I]5′-iododeoxyuridine instead of [$^3$H]thymidine to facilitate sample processing for counting. For assay, cells were seeded at 2.5 × 10$^5$ cells ml$^{-1}$ in 96-well trays and grown to confluence. After rendering the cells quiescent by incubation in 1% FBS/DMEM for 48 h, test substances (antibody, EGF, column fractions) were added to cells in serum-free DMEM–PBS (1:1) containing 1% bovine serum albumin, 4 μg ml$^{-1}$ insulin, 240 μg ml$^{-1}$ transferrin, 25 mM HEPES buffer, pH 7.4. Stimulation of DNA synthesis was assessed 23 h later by pulsing the cells for 2 h with

[$^{125}$I]5'-iododeoxyuridine (92 kBq ml$^{-1}$, 74 TBq mmol$^{-1}$) containing 5 μM 5-fluor-2'-deoxyuridine (FUdR). Acid-insoluble radioactivity was determined by gamma counting.

*Enzyme-linked immunosorbent assays (ELISAs) for anti-EGF IgG and EGF*

Sheep anti-EGF IgG was measured in sera of treated mice using EGF (1.6 pmol per well) which was immobilised onto the wells of an ELISA tray by incubation overnight in buffer containing 35 mM sodium bicarbonate, 15 mM sodium carbonate and 0.2% sodium azide, pH 9.6. After blocking residual binding sites using 1% (w/v) bovine serum albumin in 50 mM Tris-HCl, 145 mM sodium chloride, 0.05% (vlv) Tween-20, pH 7.4 (TB/BSA buffer), serum samples were diluted in the same buffer (1:5 and four serial doubling dilutions) and applied to the wells. Following a 2 h incubation at 37°C, bound antibody was detected, after washing, by incubating with 100 μl per well rabbit anti-sheep immunoglobulins conjugated to horseradish peroxidase (diluted 1:1,000) for 1 h at 37°C before visualisation using 0.01% hydrogen peroxide in 0.1 M sodium acetate buffer containing 0.015 mM 3,3',5,5'-tetramethylbenzidine dihydrochloride (TMB) as chromogen. The colour reaction was terminated by acidification (50 μl of 2 M sulphuric acid per well) and the absorbance of each well was recorded at 450 nm. Sheep anti-EGF was quantitated by reference to a standard curve generated by dilution of known quantities of the antibody (0-25 μg ml$^{-1}$). The initial dilution of antibody was prepared in sera obtained from untreated mice.

EGF was measured in urine and serum of mice using a competitive binding immunoassay in which EGF contained in samples competed with immobilised EGF for binding to an anti-EGF antibody. Sera or urine samples were diluted in TB/BSA buffer (1:1 or 1:80 respectively, and four serial doubling dilutions) in an ELISA tray and then mixed with an equal volume of rabbit anti-EGF IgG (1 μg ml$^{-1}$). After incubation overnight at 4°C, 100 μl aliquots were transferred to a second ELISA tray to which EGF has been coupled as above. Following a 2 h incubation, antibody bound to the ELISA tray was detected using swine anti-rabbit immunoglobulins conjugated to horseradish peroxidase following the procedure outlined above. EGF contained in the samples was estimated by reference to a standard curve generated using mouse EGF (0-8 nM).

EGF was measured in column fractions using a sandwich ELISA which was approximately three times more sensitive than the competitive binding assay described above. In this assay, EGF was trapped by sheep anti-EGF IgG (1.5 μg) coupled to the wells of an ELISA tray. Bound EGF was detected by rabbit anti-EGF IgG (0.5 μg per well) followed by a peroxidase-conjugated anti-rabbit IgG with colorimetric detection as above. EGF in samples was estimated by reference to a standard curve generated using mouse EGF (0-3.0 nM).

*Immunocytochemical staining of mouse kidney*

Frozen sections of kidney (approximately 5 μm thick) were fixed in dry acetone and processed using standard immunohistochemical techniques. Primary sheep anti-EGF IgG applied to tissue sections or sheep anti-EGF IgG immune complexes localised in damaged kidneys of treated mice were detected using an anti-sheep immunoglobulin-peroxidase conjugate which was visualised using hydrogen peroxide - 3,3'-diaminobenzidine (DAB) as chromogen.

### Results

*Growth of MT1 carcinoma cells in the peritoneal cavity and the effect of pristane on growth*

Following intraperitoneal injection, MT1 cells grew as multiple solid tumour nodules. The initial site of growth was the greater omentum, followed by the mesentery and fat. The diaphragm and muscle wall were colonised as a secondary event. The pattern of growth of the MT1 tumour cells suggested that the tumour deposits had originally arisen from growth of a single cell or a small cluster of cells and that the initial establishment of the tumour cell colony might thus mimic the growth of a metastatic cancer cell after it had left the circulation and finally lodged at a site distant from the primary tumour.

The number of tumour cells needed to induce solid tumours was greatly reduced in the mice injected with the mineral oil pristane, which induces a macrophage-rich ascites. Table I shows a comparison of the incidence of tumour take in relation to the number of cells injected into control mice or mice which had been injected with 0.5 ml of pristane i.p. 14 days prior to inoculation of the carcinoma cells. Whereas tumour growth was observed when $10^2$ to $10^3$ tumour cells were inoculated into control mice, tumour growth was observed using an inoculum containing only ten cells in the pristane-treated mice. Our observation of tumour growth using such a low inoculum further supports the concept that growth within the peritoneal cavity can be considered as a model for growth of isolated cancer cells. Optimum promotion of growth was obtained when 0.5 ml of pristane was injected i.p. 14 days prior to the inoculation of carcinoma cells. In contrast, no sensitisation was observed when $10^2$ tumour cells were inoculated into the peritoneal cavity 3 or 30 days after pristane treatment. At day 14, pristane was found to cause an enlargement of the omentum and increased cellularity of the omentum and mesentery. The number of cells that could be retrieved from the peritoneal cavity by lavage was also increased; the peritoneal exudate was found to contain approximately $2.2 \times 10^7$ macrophages (Table II). These effects of pristane were not observed at 3 or 30 days. These results suggest that pristane enhanced establishment and growth of MT1 cells in the peritoneal cavity by induction of an inflammatory infiltrate.

*Absence of EGF in extracts of MT1 tumours*

As EGF has been implicated in tumour development and progression, this growth factor was selected as a possible candidate molecule produced by the host which might be

**Table I** Effect of pristane on the intraperitoneal growth of MT1 tumour

| *Number of cells injected* | *Tumour prevalence* Control | *Tumour prevalence* Pristane treated |
|---|---|---|
| 10 | 0/5 | 1/5; 4/5 |
| $10^2$ | 1/5 | 5/5 |
| $10^3$ | 3/5 | 5/5 |
| $10^4$ | 5/5 | 5/5 |

Mice were treated with pristane (0.5 ml of pristane i.p.) 14 days prior to inoculation of tumour cells. Inocula containing log dilutions of MT1 cells were injected into the peritoneal cavity of five untreated or five pristane-treated mice. After 30 days, the animals were killed and tumour prevalence determined at autopsy.

**Table II** Effect of time following pristane injection on sensitisation of tumour take and number of macrophages in the peritoneal exudate

| *Duration of pristane sensitisation (days)* | *Tumour prevalence* | *Macrophages detected in peritoneal exudate* |
|---|---|---|
| Control (no pristane) | 1/5 | $0.4 \times 10^6$ |
| 3 | 0/5 | $1.2 \times 10^6$ |
| 14 | 5/5 | $2.2 \times 10^7$ |
| 30 | 1/5 | $2.1 \times 10^6$ |

Two groups, each of five mice, were treated with 0.5 ml of pristane i.p. for the times shown above. One group was then inoculated with $10^2$ MT1 cells and tumour prevalence determined 28 days later. The other group was subjected to peritoneal lavage to determine the number of cells present in the peritoneal exudate at the time of inoculation. Macrophages were identified and counted as described in the Materials and methods section.

responsible for the promotion of growth of MT1 in the peritoneal cavity. However, to be able to determine whether EGF produced by the host contributed to proliferation of MT1, it was necessary to establish that the tumour did not produce this growth factor.

Initially, MT1 tumour tissue derived from subcutaneous tumour implants was extracted into acid as previously described (Koyama & Podolsky, 1989). Separation of this extract by FPLC Mono-Q anion-exchange chromatography resulted in the detection of several peaks of mitogenic activity, however none of this activity could be blocked by anti-EGF IgG (Figure 1a), nor could immunoreactive EGF be detected in crude extracts or the individual fractions (data not shown).

As EGF is also synthesised as a high molecular weight membrane-bound precursor which has been reported to be biologically active (Mroczkowski *et al.*, 1989), detergent extracts of MT1 tumour were also analysed to test if EGF occurred in the tumour in this form. Conditions for the extraction, partial purification by gel filtration and assay of the 150 kDa membrane-bound EGF precursor were established using mouse kidney. Even though kidney contains substantially lower levels of EGF than salivary glands, the developed procedure enabled detection of both the precursor and mature forms of EGF using both a sensitivie ELISA and the mitogenic assay of growth-arrested human foreskin fibroblasts (data not shown). Analysis of detergent extracts of MT1 tumours by this procedure yielded fractions which demonstrated mitogenic activity in the standard assay; however, as found with the acidic extracts, the activity of these fractions could not be blocked by anti-EGF antibody (Figure 1b), nor could immunoreactive EGF be detected by ELISA. Moreover, the mitogenic activity was not blocked by an antibody directed against the EGF receptor (EGFR) (Figure 1b), which blocks the mitogenic activity of both EGF and TGF-α. The nature of the mitogenic activity found in the extracts of MT1 cells remains to be elucidated, but it was neither EGF nor its membrane-bound high molecular weight precursor. Given the lower limit of detection of our assays, we calculate that MT1 tumour can only contain less than 1 pmol of EGF per g wet weight of tumour tissue.

### *Effect of EGF on growth of MT1 cells* in vitro

Having established the absence of EGF in MT1 tumours, the effect of EGF on *in vitro* growth of MT1 cells was investigated. MT1 cells, obtained by proteolytic disaggregation from the tumour, were readily established *in vitro* in 10% FBS/DMEM. The cells were adherent and exhibited cuboidal and fusiform morphologies as previously reported (Barnett & Eccles, 1984). The MT1 cells could not be grown in serum-free medium even when this had been supplemented with insulin and transferrin, but could be grown in medium containing serum which had been depleted of cationic growth factors such as platelet-derived growth factor (PDGF) by passage over a cation-exchange resin (cNBS). When MT1 cells were cultured in 2% cNBS/DMEM, 1 nM EGF stimulated growth as evidenced by an increase in cell number (Figure 2). The most marked effect of EGF was to reduce the lag phase preceding growth; once the cells had established growth, EGF provided no additional stimulus.

### *Inhibition of mitogenic activity of EGF by anti-EGF IgG and correlation with serum sheep anti-EGF IgG levels*

To determine whether host-derived EGF contributed to establishment of MT1 tumours grown in the peritoneal cavity, the effect of an anti-EGF IgG on tumour growth was determined. The IgG preparation used in the investigations described below was first tested for its ability to inhibit EGF-induced DNA synthesis. The mitogenic action of EGF was assayed by the standard procedure of initiating DNA synthesis in growth-arrested cultures of human foreskin fibroblasts. Figure 3 shows the degree of inhibition produced by increasing doses of the antibody on the stimulation of DNA synthesis induced by 1 nM EGF. Inhibition was complete using immune IgG at a concentration of $10\,\mu g\,ml^{-1}$, whereas similar doses of control, non-immune, IgG had no effect on EGF-stimulated DNA synthesis. The anti-EGF antibody produced no inhibition of mitogenesis induced by TGF-α (see Figure 3), even though these two ligands are structurally homologous and both bind to the EGF receptor. Table III shows the range of concentrations of EGF over which a dose of $380\,\mu g\,ml^{-1}$ antibody is effective.

The concentration of sheep anti-EGF IgG in serum of mice treated with a single i.p. injection of 2.5 mg antibody was assessed by ELISA. The level of sheep anti-EGF IgG attained a peak serum level *in vivo* of $718\,\mu g\,ml^{-1}$ within 1 h of administration and decayed to $230-240\,\mu g\,ml^{-1}$ over 4–5 days (Table IV). Figure 3 shows that, in an *in vitro* assay, as little as $10\,\mu g\,ml^{-1}$ antibody is capable of inhibiting completely the mitogenic activity of 1 nM EGF, and Table III shows that, at $380\,\mu g\,ml^{-1}$, the antibody is able to inhibit

**Figure 2** Effect of EGF on growth of MT1 cells *in vitro*. MT1 cells were plated at $0.5 \times 10^5$ cells $ml^{-1}$ in the presence (▲) or absence (Δ) of 1 nM EGF. At appropriate times, duplicate flasks of cells were harvested and viable cells determined by trypan blue exclusion and counting. The results show the mean cell number determined at each time point.

**Figure 3** Inhibition of EGF-induced DNA synthesis by sheep anti-EGF IgG. Stimulation of DNA synthesis in confluent and quiescent human foreskin fibroblasts was measured in response to 1 nM EGF (□) or TGF-α (Δ) as described in the Materials and methods section. The figure shows the effect of the presence of increasing concentrations of sheep anti-EGF IgG (open symbols) or IgG prepared from an unimmunised sheep (closed symbols) on this stimulation. The results are representative of two individual experiments.

**Table III** Inhibition of EGF-induced DNA synthesis in human foreskin fibroblasts by sheep anti-EGF IgG

| [EGF] (nM) | Inhibition of DNA synthesis (%) |
|---|---|
| 2 | 88 |
| 8 | 85 |
| 32 | 80 |
| 128 | 24 |
| 512 | 0 |
| 2048 | −3 |

The ability of a fixed dose of sheep anti-EGF IgG (380 μg $ml^{-1}$) to inhibit DNA synthesis in response to increasing doses of EGF was assayed using quiescent human foreskin fibroblasts in a standard mitogenesis assay as described in the Materials and methods section. Results are expressed as percentage inhibition of DNA synthesis when compared with duplicate wells of cells treated with each dose of EGF alone. In the absence of antibody, 2 nM EGF produced maximal stimulation of DNA synthesis, i.e. the doses of EGF tested were all in excess of that required to saturate the system.

**Table IV** Serum antibody and urinary EGF levels in mice treated with a single dose of sheep anti-EGF IgG

| Time after administration of antibody (h) | Sheep anti-EGF IgG in serum (μg $ml^{-1}$) | Urinary EGF (μg $ml^{-1}$) |
|---|---|---|
| 0 | – | 4.4 |
| 1 | 718 | 4.7 |
| 6 | 640 | 15.4 |
| 18 | 384 | 14.1 |
| 24 | 272 | 9.4 |
| 50 | 240 | 17.1 |
| 105 | 234 | 15.3 |

Mice were treated with 2.5 mg of sheep anti-EGF IgG intraperitoneally. At each time point, three animals were killed and serum and urine collected and combined for determination of sheep anti-EGF IgG and EGF respectively, as described in the Materials and methods section.

**Table V** Effect of treatment with anti-EGF IgG on intraperitoneal growth of MT1 cells

| Tumour prevalence | |
|---|---|
| Control (five mice) | Anti-EGF IgG (five mice) |
| Two + + | Three none |
| Three + + + | One + |
| | One + + |

Approximately $5 \times 10^3$ mammary carcinoma cells were transplanted into the peritoneal cavity of mice, which were then either left untreated or treated with 2.5 mg of sheep anti-EGF intraperitoneally three times per week, starting 2 days after transplantation. After 29 days, animals were killed and tumour burden assessed as follows: +, <10 small nodules confined to omentum; + +, omentum extensively involved, no spread; + + +, widespread involvement including muscle wall.

concentrations of up to 32 nM EGF; for comparison, the serum level of EGF of untreated mice was less than the lowest level which our ELISA allowed us to determine, which was 0.12 nM. It is clear, therefore, that the circulating level of antibody was greatly in excess of EGF in the circulation.

*Treatment of MT1 carcinoma for 29 days with anti-EGF IgG*

In a preliminary experiment to determine the effect of anti-EGF IgG on growth of MT1, two groups, each of five mice, were inoculated i.p. with $5 \times 10^3$ MT1 tumour cells; one group was given 2.5 mg of sheep anti-EGF IgG i.p. on alternate days starting 3 days after inoculation of tumour cells and the second group was left untreated. On day 29 of the experiment the first mouse in the control group was becoming moribund and the experiment was terminated at this time. All of the mice were killed and autopsied. The findings are summarised in Table V and demonstrate that the antibody exerted a pronounced growth-inhibitory effect.

**Figure 4** Anti-tumour effect of long-term treatment with sheep anti-EGF IgG. Approximately $5 \times 10^3$ MT1 tumour cells were transplanted into the peritoneal cavity of mice, which were then either left untreated ( ■ ) or treated with 2.5 mg of non-immune sheep IgG ( □ ) or anti-EGF IgG ( ▨ ) using the regimen described in the legend to Table V. The chart shows the times at which individual animals in each group were killed because of tumour burden. Analysis of the data using Wilcoxon's rank correlation test showed that there was a significant difference between survival of either untreated or control IgG-treated animals *vs* anti-EGF IgG-treated animals ($P<0.05$).

*Anti-tumour effect of long-term treatment with anti-EGF IgG*

Three groups of ten mice, inoculated with MT1 tumour cells as in the preceding experiment, received either (a) no treatment, (b) normal IgG from an unimmunised sheep or (c) sheep anti-EGF IgG. Three days after inoculation of the tumour cells, 2.5 mg of the immunoglobulins were injected into the peritoneal cavity on alternate days for 4 weeks. Unlike the preliminary experiment, survival of each individual mouse was followed. The experiment was terminated after 110 days when surviving animals were killed and autopsied. The outcome of the experiment is shown in Figure 4. There was no significant difference in the rate of tumour development between the groups receiving no treatment or control IgG from a non-immunised sheep. In these two groups, morbidity from tumour burden first occurred at days 30 and 32 respectively and eight out of ten animals had to be killed as result of tumour growth by day 60. The remaining two animals in both of the groups were alive and free of tumours when killed 3 months later. In the group of mice which received the anti-EGF IgG, three animals in the group were killed between days 60 and 67 as a result of tumour growth; the remaining seven animals had to be killed because of their poor condition between days 83 and 95, but on autopsy no tumour could be detected and morbidity was found to be due to renal failure. Indeed, the kidneys were extensively damaged in all ten mice in this group, including the three that at autopsy contained some tumour.

Histochemical examination of the damaged kidneys showed that the lesions were due to glomerular deposition of immune complexes containing sheep IgG; no such immune complex disease was found in mice that had received the non-immune sheep IgG (results not shown). When normal kidney sections were stained with the anti-EGF IgG, staining was seen in the distal tubules which have previously been found to be a site of EGF synthesis by *in situ* hybridisation (Salido *et al.*, 1989). These results suggest that the cause of the kidney damage was not due to the reaction of the antibody with EGF produced in the kidneys, as this occurs at a different site (i.e. the distal tubules), but rather from the immune complex deposition in the glomeruli.

Although anti-EGF IgG treatment caused long-term kidney damage, the antibody had no immediate effect on the production of EGF by the distal tubules in short-term experiments in which output of EGF in the urine was measured following a single i.p injection of 2.5 mg of antibody. Table IV shows that anti-EGF IgG treatment did not cause the amount of EGF in the urine over a 7 day period to fall. Since in these animals antibody was present in great excess, EGF in the urine is unlikely to be derived to any significant extent from EGF circulating in the blood or extracellular tissue fluids. Unfortunately, no urine was taken after prolonged antibody treatment which gave rise to the lethal kidney lesions. Our results support the findings of Mattila *et al.* (1988), who concluded that urinary EGF in humans has a renal origin.

*Response of MT1 carcinoma cells growing subcutaneously*

To compare the effect of anti-EGF IgG treatment on the growth of MT1 cells at a site other than the peritoneal cavity, MT1 cells were grown subcutaneously. The cell dose needed to induce a tumour at a subcutaneous site of injection was found to be much greater than that needed for tumour growth intraperitoneally. Inoculation of $10^4$ cells caused tumours in two out of five mice, whereas inoculation of $5 \times 10^4$ cells caused tumours in five out of five mice. After ten mice were inoculated with $5 \times 10^4$ cells, five were treated with the anti-EGF antibody by the protocol used in the preliminary (i.e. 29 day) treatment of tumours and five mice were left untreated. Both treated and control groups had 100% tumour prevalence and there was no significant difference in the size of the tumour in the control and treated groups. The tumour weight was, on average, 0.45 g in the control and 0.49 g in the treated group.

### Discussion

The present investigation has demonstrated that host-derived factors can influence the growth of the MT1 mammary carcinoma when transplanted into the peritoneal cavity. In this environment the tumour grows as multiple solid nodules which have probably arisen from individual cells. In this respect, factors influencing the initial establishment of MT1 tumour microcolonies may be similar to those that influence the ability of a metastatic cancer cell to grow once it has left the circulation and lodged at a distant site in the body. In previous studies, it has been shown that blood-borne cancer cells exhibit organ-selective tumour growth (Murphy *et al.*, 1988). Similarly, tumour outgrowth has been shown to occur preferentially at sites of wound healing, and it has been postulated that factors released by host cells into the inflammatory wound environment to promote healing also facilitate tumour growth (Skipper *et al.*, 1988). In the present study, the growth-promoting properties of pristane correlated with its ability to produce an inflammatory environment within the peritoneal cavity, and here again enhanced tumour take is likely to be due to direct or indirect effects of host-derived growth factors or cytokines released at the inflammatory site.

One of the factors found to contribute to tumour growth was identified as EGF on the basis of the anti-tumour effect of an anti-EGF antibody which only inhibits EGF and does not cross-react with other growth factors such as TGF-α or PDGF. As the MT1 carcinoma was shown not to produce any detectable EGF, the inhibition of its growth by antibody could not, therefore, be ascribed to interference of autocrine growth stimulation. Although we cannot completely exclude the possibility that the kidney damage caused by long-term anti-EGF treatment accounted for the decreased tumour growth, this seems unlikely as in the initial short-term experiment in which animals were followed for up to 28 days no evidence of kidney damage was evident at autopsy even though tumour growth was reduced by the antibody treatment.

EGF may have caused stimulation of growth of MT1 through a number of mechanisms. One possible interpretation of our results is that host-derived EGF has a direct effect on growth of MT1 cells *in vivo* and that this gives them an initial growth advantage. At this time, when the tumour cells are spread within the peritoneal cavity and isolated from each other, diffusion of autocrine factors away from the cell surface may limit their availability and, similarly, availability of membrane-bound factors would be limited only to cells in direct contact. This proposal is consistent with the observed ability of EGF to reduce the time taken before establishment of log phase growth of MT1 *in vitro*. Alternatively, a more complex situation may exist whereby EGF exerts indirect effects on growth of MT1. For example, depletion of host-derived EGF may prevent induction of other growth factors or cytokines which facilitate tumour growth or which are responsible for induction of the inflammatory environment. In this context, it has been shown that EGF and TGF-α regulate cytokine production by human thymic epithelial cells (Le *et al.*, 1991).

The host-derived EGF which promotes the growth of the carcinoma cells may come from circulating EGF in the blood, however the plasma concentration of EGF is extremely low. Since, as shown in Table I, growth of MT1 cells is greatly facilitated within a peritoneum enriched in leucocytes as a result of prior stimulation by pristane, the EGF involved may come from this source. Platelets contain a high concentration of EGF which is released on clotting (Nakamura *et al.*, 1989; Hwang *et al.*, 1992). Although activated macrophages have been shown to synthesise TGF-α (Madtes *et al.*, 1988), whether they produce EGF has not been clearly established. They do however, produce a related form of EGF, namely heparin-binding EGF (Higashiyama *et al.*, 1991); cross-reactivity of our antibody with this ligand, or with other EGF receptor ligands such as amphiregulin (Shoyab *et al.*, 1989), cannot be excluded until authentic forms of the murine ligands are available. Alternatively, a more complex paracrine mechanism may be operative whereby growth factors (such as the colony-stimulating factors) which are produced by macrophages up-regulate expression of EGF by fibroblasts or epithelial cells in the peritoneal cavity and this is then available to promote growth of the MT1 tumour cells.

In contrast to the results obtained with MT1 grown intraperitoneally, subcutaneous growth of MT1 was not affected by anti-EGF IgG treatment. The failure of the antibody to affect the growth of MT1 cells growing subcutaneously is unlikely to be due to limited access of antibody delivered to the subcutaneous site. If EGF exerted a direct effect on intraperitoneal growth of MT1, then the differing results may be explained by the relatively large numbers of cells which were necessary for subcutaneous growth of the tumour (which developed as a single discrete nodule); it is possible that the large inoculum enabled establishment of a microenvironment which facilitated proliferation, possibly by an autocrine mechanism involving growth factors produced by the MT1 cells themselves. Alternatively, if EGF exerted an indirect effect on intraperitoneal growth of MT1, the different response of the subcutaneous tumour to antibody treatment may be accounted for by the requirement for EGF to promote the formation of an inflammatory environment within the peritoneal cavity.

Extensive studies examining the effect of anti-human EGF receptor antibodies (which do not recognise murine EGF receptors) on the growth of human tumours grown as xenografts in nude mice have indicated their growth-inhibitory potential, particularly when used in combination with cytotoxic drugs (Baselga *et al.*, 1993). The involvement of host-derived EGF in facilitating establishment of microcolonies of MT1 tumour cells may indicate further new directions for carcinoma therapy. Although the results of this study demonstrate an anti-carcinoma effect of anti-EGF antibody, such an antibody approach has no therapeutic value for it induces severe kidney damage. Although EGF receptor antibodies have been administered as single doses to patients for tumour imaging (Divgi *et al.*, 1991), the long-term toxicity of repeated administration of such antibodies to human subjects remains to be determined (see Baselga *et al.*, 1993).

This work was supported by the Cancer Research Campaign, UK.

### References

ALEXANDER, P. (1987). The role of the host in facilitating and controlling the development and spread of cancer. In *Biology of Carcinogenesis*, Waring, M.J. & Ponder, B. (eds) pp. 191-221. Lancaster: MTP Press.

BARNETT, S.C. & ECCLES, S.A. (1984). Studies of mammary carcinoma metastasis in a mouse model system. I. Derivation and characterization of cells with different metastatic properties during tumour progression *in vivo*. *Clin. Exp. Metastasis*, **2**, 15-36.

BASELGA, J., NORTON, L., MASUI, H., PANDIELLA, A., COPLAN, K., MILLER, W.H. & MENDELSOHN, J. (1993). Antitumour effects of doxorubicin in combination with anti-epidermal growth factor receptor monoclonal antibodies. *J. Natl Cancer Inst.*, **85**, 1327-1333.

BLACKLER, J., ALEXANDER, P., FARMER, S. & DAVIES, D.E. (1988). Effect of anti-EGF antibody on cell growth *in vitro* and tumour growth *in vivo*. *Br. J. Cancer*, **57**, 223-224.

BREILLOUT, F., ANTOINE, E., LASCAUX, V., ROLLAND, Y. & POUPON, M.-F. (1989). Promotion of micrometastasis proliferation in a rat rhabdomyosarcoma model by epidermal growth factor. *J. Natl Cancer Inst.*, **81**, 703-705.

CARPENTER, G. & COHEN, S. (1976). Human epidermal growth factor and the proliferation of human fibroblasts. *J. Cell Physiol.*, **88**, 227-238.

DIVGI, C.R., WELT, S., KRIS, M., REAL, F.X., YEH, S.D.J., GRALLA, R., MERCHANT, B., SCHWEIGHART, S., UNGER, M., LARSON, S.M. & MENDELSOHN, J. (1991). Phase I and imaging trial of indium 111-labelled anti-epidermal growth factor receptor monoclonal antibody 225 in patients with squamous cell lung cancer. *J. Natl Cancer Inst.* **83**, 97-104.

GINSBURG, E. & VONDERHAAR, B.K. (1985). Epidermal growth factor stimulates the growth of A431 tumours in athymic mice. *Cancer Lett.*, **28**, 143-150.

HIGASHIYAMA, S., ABRAHAM, J.A., MILLER, J., FIDDES, J.C., KLAGSBRUN, M. (1991). A heparin-binding growth factor secreted by macrophage-like cells that is related to EGF. *Science*, **251**, 936-939.

HWANG, D.L., LEV-RAN, A., YEN, C.F. & SNIECINSKI, I. (1992). Release of different fractions of epidermal growth factor from human platelets *in vitro*: preferential release of 140 kDa fraction. *Regul. Pept.*, **37**, 95-100.

INUI, T., TSUBURA, A. & MORII, S. (1989). Incidence of precancerous foci of mammary glands and growth rate of transplantable mammary cancers in sialoadenectomized mice. *J. Natl Cancer Inst.*, **81**, 660-663.

KOYAMA, S. & PODOLSKY, D.K. (1989). Differential expression of transforming growth factors α and β in rat intestinal epithelial cells. *J. Clin. Invest.*, **83**, 1768-1773.

KURACHI, H., OKAMOTO, S. & OKA, T. (1985). Evidence for the involvement of the submandibular gland epidermal growth factor in mouse mammary tumorigenesis. *Proc. Natl Acad. Sci. USA*, **82**, 5940-5943.

LE, P.T., LAZORICK, S., WHICHARD, L.P., HAYNES, B.F. & SINGER, K.H. (1991). Regulation of cytokine production in the human thymus: epidermal growth factor and transforming growth factor α regulate mRNA levels of interleukin 1α (IL-1α) IL-β, and IL-6 in human thymic epithelial cells at a post-transcriptional level. *J. Exp. Med.*, **174**, 1147-1157.

MADTES, D.K., RAINES, E.W., SAKARIASSEN, K.S., ASSOIAN, R.K., SPORN, M.B., BELL, G.I. & ROSS, R. (1988). Induction of transforming growth factor-α in activated human alveolar macrophages. *Cell*, **53**, 285-293.

MATTILA, A.-L., VIINIKKA, L., SAARIO, I. & PERHEENTUPA, J. (1988). Human epidermal growth factor: renal production and absence from plasma. *Regul. Pept.*, **23**, 89-93.

MROCZKOWSKI, B., REICH, M., CHEN, K., BELL, G.I. & COHEN, S. (1989). Recombinant human epidermal growth factor precursor is a glycosylated membrane protein with biological activity. *Mol. Cell. Biol.*, **9**, 2771-2778.

MURPHY, P., ALEXANDER, P., SENIOR, P.V., FLEMING, J., KIRKHAM, N. & TAYLOR, I. (1988). Mechanisms of organ selective tumour-growth by bloodborne cancer cells. *Br. J. Cancer*, **57**, 19-31.

NAKAMURA, T., KASAI, K., BANBA, N., ISHIKAWA, M. & SHIMODA, S.-I. (1989). Release of human epidermal growth factor from platelets in accordance with aggregation *in vitro*. *Endocrinol. Jpn.*, **36**, 23-28.

OZAWA, S., UEDA, M., ANDO, N., ABE, O., HIRAI, M. & SHIMIZU, N. (1987). Stimulation by EGF of the growth of EGF receptor-hyperproducing tumor cells in athymic mice. *Int. J. Cancer*, **540**, 706-710.

SALIDO, E.C., YEN, P.H., SHAPIRO, L.J., FISHER, D.A. & BARAJAS, L. (1989). In situ hybridization of prepro-epidermal growth factor mRNA in the mouse kidney. *Am. J. Physiol.*, **256**, F632-F638.

SHOYAB, M., PLOWMAN, G.D., McDONALD, V.L., BRADLEY, J.D. & TODARO, G.J. (1989). Structure and function of human amphiregulin: a member of the epidermal growth factor family. *Science*, **243**, 1074-1076.

SINGLETARY, S.E., FRAPPAZ, D., TUCKER, S.L., LARRY, L., BROCK, W.A. & SPITZER, G. (1989). Epidermal growth factor effect on serum-free growth of primary and metastatic human tumours. *Int. J. Cell Cloning*, **7**, 59-66.

SKIPPER, D., JEFFREY, M.J., COOPER, A.J., TAYLOR, I. & ALEXANDER, P. (1988). Preferential growth of bloodborne cancer cells in colonic anastomoses. *Br. J. Cancer*, **57**, 564-568.

SPORN, M.B. & TODARO, G.J. (1980). Autocrine secretion and malignant transformation of cells. *N. Engl. J. Med.*, **303**, 878-880.

TSUTSUMI, O., TSUTSUMI, T. & OKA, T. (1987). Importance of epidermal growth factor in implantation and growth of mouse mammary tumour in female nude mice. *Cancer Res.*, **47**, 4651-4653.